# Self-reported symptoms of depression and anxiety among informal caregivers of persons with dementia: a cross-sectional comparative study between Sweden and Italy

**DOI:** 10.1186/s12913-020-05964-2

**Published:** 2020-12-02

**Authors:** Joseba Wulff, Agneta Malmgren Fänge, Connie Lethin, Carlos Chiatti

**Affiliations:** 1 Department of Health Sciences, Faculty of Medicine, Lund University, Box 157, SE-221 00 Lund, Sweden; 2 Department of Clinical Sciences, Clinical Memory Research Unit, Faculty of Medicine, Lund University, SE-214 28 Malmö, Sweden

**Keywords:** Anxiety, Dementia, Depression, Informal caregivers, Italy, Regression analysis, Sweden

## Abstract

**Background:**

Around 50 million people worldwide are diagnosed with dementia and this number is due to triple by 2050. The majority of persons with dementia receive care and support from their family, friends or neighbours, who are generally known as informal caregivers. These might experience symptoms of depression and anxiety as a consequence of caregiving activities. Due to the different welfare system across European countries, this study aimed to investigate factors associated with self-reported depression and anxiety among informal dementia caregivers both in Sweden and Italy, to ultimately improve their health and well-being.

**Methods:**

This comparative cross-sectional study used baseline data from the Italian UP-TECH (*n* = 317) and the Swedish TECH@HOME (*n* = 89) studies. Main outcome variables were the severity of self-reported anxiety and depression symptoms, as measured by the Hospital Anxiety and Depression Scale (HADS). HADS scores were investigated using descriptive and bivariate statistics to compare means and standard deviations. Linear regressions were used to test for associations between potential factors and self-reported symptoms of depression and anxiety.

**Results:**

Italian informal caregivers reported more severe symptoms of depression and anxiety than Swedish caregivers. In Italy, a higher number of hours of caregiving was associated with anxiety symptoms (β = − 1.205; *p* = 0.029), being 40–54 years-old with depression symptoms (β = − 1.739; *p* = 0.003), and being female with symptoms of both depression (β = − 1.793; *p* < 0.001) and anxiety (β = 1.474; *p* = 0.005). In Sweden, a higher number of hours of caregiving and being < 39 years-old were associated with depression symptoms (β = 0.286; *p* < 0.000; β = 3.945; *p* = 0.014) and a higher number of hours of caregiving, the lack of additional informal caregivers and dementia severity were associated with anxiety symptoms (β = 0.164; *p* = 0.010; β = − 1.133; *p* = 0.033; β = − 1.181; *p* = 0.031).

**Conclusion:**

Multiple factors are associated with self-reported symptoms of depression and anxiety among informal caregivers in Sweden and Italy. Factors found in this study partly differ between the two countries, suggesting the important role of cultural and social factors affecting the experience of caregiving. A deeper knowledge of these factors may increase the knowledge on potential protective and risk factors, provide information to policymakers and ultimately improve the psychological well-being of informal caregivers to people with dementia across Europe.

## Background

Dementia is a disease with a complex aetiology that impairs cognitive function, leading to a deterioration of memory, physical, emotional and behavioural control [1, 2]. There are approximately 50 million persons living with dementia worldwide and this number is bound to triple by 2050, due to the growing older population. Most persons with dementia are aged 65 years or older; however, an estimated 6–9% of all cases are persons with young-onset dementia [2, 3]. In 2018, the global societal cost of dementia was about US$ 1 trillion, mainly within high-income countries (HIC) [4]. Out of this, around 80–85% were estimated to be associated with the costs of social care as well as informal care, including indirect costs for reduced quality of life and hours of work lost by informal caregivers [5]. Undoubtedly, dementia is a major public health concern affecting not only persons with dementia, but also their informal caregivers and the overall society and economy.

People with dementia experience symptoms like memory loss, confusion, change in personality, and difficulties with activities of daily living (ADL), such as washing or cooking. These symptoms may become problematic as they advance in severity throughout the disease, including the early, middle and late-stage; lasting from several months to years. Symptoms may be managed by pharmaceutical treatment and support provided by healthcare and social services [2]. However, due to cultural norms and difficulties with accessibility and affordability, people with dementia in Europe vary in dependency on care and support by their families, friends and neighbours [6], i.e. the informal caregivers (referred to as ‘caregivers’ in this paper) [7].

Whilst in some cases caregivers experience positive effects in their caregiving role, often the effects are perceived negatively. Since caregivers may experience various forms of physical, financial, social or psychological challenges, they are at higher risk of subjective caregiver burden [8]. Caregivers of persons with dementia have shown to be at higher risk of experiencing symptoms of depression and anxiety compared to caregivers of persons with other illnesses [9]. They may lose the capacity to care for persons with dementia and their own health [10, 11]. Thus, especially in a time when the demand for caregivers is expected to increase with the rising number of people with dementia worldwide, it is particularly important to investigate further what can be done to tackle this public health issue.

The WHO recommends that each country should have a specific action plan to improve “person-centred, gender-sensitive, culturally appropriate care” for persons with dementia and their caregivers [12]. Even though different intervention strategies that alleviate depression and anxiety symptoms have proven successful, the most effective approach has not been fully identified. This might include multicomponent approaches, as well as tailored-made interventions that target the caregiver’s needs; more specific training, teaching programs, support groups, direct assistance, psychosocial interventions, and technology-based programs [13–15].

Conversely, research has shown that specific factors increase the risk of symptoms of depression or anxiety among caregivers of persons with dementia. These factors include caregivers being female [16, 17]; or being the persons with dementia’s spouse [10]; having unhealthy personality traits, like low confidence in the caregiver role [8, 17]; low-level education [18]; poor physical health [19, 20]; or a history of psychiatric disease [21]. Caregivers that provide a substantial number of hours of caregiving per day [18]; have an occupation in the labour market [21]; have no additional support [21, 22]; have reduced time for leisure and social life [18, 23], or have a poor quality of the relationship with the persons with dementia [17, 24] may also be at increased risk. If the persons with dementia show high dependency in ADL, inappropriate behaviours [16, 25] and increased severity and symptoms of dementia [10, 16, 26], a caregiver is also more likely to develop symptoms of depression and anxiety. Age is also a factor; however, it is still unclear whether being above 65 years of age [18, 19] or younger increases the risk of experiencing symptoms of depression and anxiety [27]. The findings of these multiple studies can be summarised by the Pearlin and colleagues’ model [11] whereby: 1) background and contextual circumstances of caregivers affect 2) primary and secondary stressors of dementia, including intrapsychic stressors of the caregivers, which are regulated by 3) mediators of stress that either worsen or improve the 4) outcomes of caregiving stress, including depression and anxiety.

Even though numerous predictors of caregiver’s wellbeing when caring for a person with dementia have been investigated, findings are often inconsistent across countries, most likely due to heterogeneity in terms of population size, culture of care and healthcare and social services systems. Taking the two HIC Sweden and Italy as an example, there are around 10 million people living in Sweden, of which 20% are aged 65 years and older. Italy has a population of 60 million, of which 23% are aged 65 years or older [28, 29]. In Sweden about 18 people per 1000 population (all ages) are living with dementia and in Italy about 23 per 1000 population (all ages), being the second highest across the Organisation for Economic Co-operation and Development (OECD) [30]. However, the access and availability of health and social services for persons with dementia and intervention programs for caregivers vary considerably between Sweden and Italy. This is reflected by their differing welfare states, whereby Sweden, with its social democracy regime, strives for “equality of highest standards, rather than equality of minimal needs” as seen by Italy’s corporatist-subsidiarity regime, including rights, access to and benefits of provided healthcare [31]. In 2018, the total health care expenditure amounted to 11% of gross domestic product (GDP) in Sweden, compared to 8.8% in Italy [32]. Findings from Chiatti et al. [33] highlighted that day care centres, social services and home care were used more often in Sweden among caregivers and persons with dementia, compared to Italy. Likewise, Swedish municipalities occasionally provide financial aid to caregivers, described as ‘relative-care employment’ [34]. Contrarily, Italians with dementia receive most support from privately paid home services and family care, of which most of the formal healthcare and social services are either unavailable or not affordable [33]. Italian caregivers are often in financial strain, as most undergo out-of-pocket payments [35]. Having a corporatist-subsidiarity regime and being highly influenced by religion and traditional family-hood, it is still deeply embedded in the Italian culture, the idea that healthier members of the family bear full responsibility for the provision of care to the frailer ones. Especially women take up this normative role as a caregiver, without receiving any professional support or financial subsidies [31, 36]. Not surprisingly, a comparative analysis of dementia care organization between Sweden and Italy found that, on average, Italian caregivers provide a dramatic number of caregiving hours per day compared to their Swedish counterparts [33].

Only a few cross-national studies have been carried out in Europe specifically investigating caregiver burden, and even fewer of these studies had a comparative research approach with respect to self-reported symptoms of depression and anxiety among caregivers [22, 37]. To contribute to fill this evidence gap, we have carried out an investigation addressing the following two research questions:

1. What are the factors associated with self-reported symptoms of depression and anxiety among informal caregivers of persons with dementia across Sweden and Italy?
2. Are there any significant differences between Sweden and Italy concerning factors associated with self-reported symptoms of depression and anxiety among informal caregivers?

## Methods

This was a comparative cross-sectional study with baseline data from the UP-TECH (NCT01700556) [38] and TECH@HOME (NCT02733939) [39] studies, carried out in Sweden (2016–2019) and Italy (2013–2015) respectively.

The main objectives of UP-TECH were to investigate whether the combination of information and communication technology (ICT), case management and home visits by dementia nurses could reduce caregiver burden and the time of institutionalization among people with Alzheimer disease (AD) [38]. Similarly, the TECH@HOME project [39] aimed to investigate whether the use of ICT reduced the need for supervision of persons with dementia and thus caregiver burden. In both studies, persons with dementia and caregivers were recruited in dyads, thanks to the collaboration of the local memory clinics (Ängelholm, Skåne County, Sweden; Ancona, Pesaro, Macerata, Fermo and San Benedetto del Tronto, Marche Region, Italy) who screened their patient’s population for potential study participants. Inclusion and exclusion criteria for both studies were similar. UP-TECH required the participants to have a diagnosis of AD at intermediate stage based on the National Institute on Ageing-Alzheimer’s Association (NIA-AA); an MMSE score between 10 and 20; living in the community and having at least one informal caregiver [38, 40]. Similarly, in the TECH@HOME study, the dyads were included if the person with dementia had a diagnosis of a major neurodegenerative disorder of mild to moderate severity based on the Diagnostic and Statistical Manual of Mental Disorders (DSM-5) [41]; a score between 14 and 24 on the Mini-Mental State Examination Swedish Revision (MMSE-SR); a score between 1 and 5 on the Global Deterioration Scale (GDS) [42]; and Swedish speaking [39]. The exclusion criteria for both studies were: not providing informed consent; not being able to perform ADL; life expectancy shorter than 6 months; being involved in another trial; aged under 18 years; having ‘unstable chronic conditions’, and being close to moving away from the study location (TECH@HOME). People who were unwilling to use technological devices, required institutionalised care, or had a substance use disorder based on the DSM-5 (UP-TECH) were excluded [38, 39]. A total of 89 Swedish and 317 Italian dyads were included in this study (Table 1). We anticipated that the final sample size was sufficient to detect a difference of at least 1.68 points between equal size groups (considering a SD equal to 4, as consistently recurring in literature) with a power of 80% and a level of significance of 0.05 [43–45].

**Table 1:** Baseline characteristics of persons with dementia and informal caregivers in Sweden and Italy

Persons with dementia			Informal caregiver		
**Characteristicss**	**Country**		**Characteristics**	**Country**	
	**Italy (*n*** **= 317)** ***N*** **(%)**	**Sweden (*n*** **= 89)** ***N*** **(%)**		**Italy (*n*** **= 317)** ***N*** **(%)**	**Sweden (*n*** **= 89)** ***N*** **(%)**
**Gender**			**Gender** ^**c**^		
Male	96 (30.3)	24 (27.0)	Male	99 (31.2)	42 (47.7)
Female	221 (69.7)	65 (73.0)	Female	218 (68.8)	46 (52.3)
**Age (years)**			**Age (years)** ^**c**^		
< 69	11 (3.5)	10 (11.2)	< 39	7 (2.2)	5 (5.7)
70–79	103 (32.5)	35 (39.3)	40–54	117 (36.9)	21 (23.9)
80–89	188 (59.3)	40 (44.9)	55–69	99 (31.2)	35 (39.8)
> 90	15 (4.7)	4 (4.5)	> 70	94 (29.7)	27 (30.7)
**Living situation**			**Living with persons with dementia** ^**c**^		
Living alone	48 (15.1)	42 (47.2)	No	114 (36.0)	48 (54.5)
Spouse	159 (50.2)	44 (49.4)	Yes	200 (63.1)	40 (45.5)
Child	68 (21.5)	1 (1.1)	**Relationship with persons with dementia**		
Other	42 (13.2)	2 (2.2)	Spouse	98 (30.9)	38 (43.2)
**Severity of disease, MMSE** ^**a**^			Child	173 (54.6)	44 (50.0)
Normal (25–30)	2 (0.6)	1 (1.2)	Other	46 (14.5)	6 (6.8)
Mild (21–24)	21 (6.6)	30 (36.1)	**Marital status** ^**c**^		
Moderate (10–20)	294 (92.7)	52 (62.7)	Married	253 (79.8)	66 (75.0)
**Inappropriate behaviours** ^**b**^			Not Married	64 (20.2)	22 (25)
No	236 (74.4)	56 (71.8)	**Education** ^**c**^		
Yes	81 (25.6)	22 (28.2)	Elementary School	186 (58.7)	43 (48.9)
**ADL dependency**			Gymnasium/ Secondary	107 (33.8)	32 (36.4)
0–2	258 (81.4)	87 (97.8)	University	24 (7.6)	13 (14.8)
3–6	59 (18.6)	2 (2.2)	**Occupation** ^**d**^		
			Not actively working	171 (53.9)	43 (50.0)
			Actively working	146 (46.1)	43 (50.0)
			**Disease count** ^**c**^	1.09 ± 1.31	0.88 ± 0.99
			**Additional caregiver support** ^**c**^		
			No additional help	60 (18.9)	44 (50.0)
			Yes additional help	257 (81.1)	44 (50.0)
			**Number of additional caregivers** ^**c**^	1.15 ± 0.86	0.97 ± 1.23
			**Total hours of caregiving/ day** ^**d**^	6.28 ± 6.44	3.67 ± 4.11

Data source for Italy: UP-TECH questionnaire; Sweden: TECH@HOME questionnaire. A primary focus was given to the informal caregivers as a variety of different characteristics was investigated for them when compared to persons with dementia.

Valid percentages (%) are provided with respect to the missing values.

*n* number of participants, N frequency value and their respective percentages or mean ± standard deviation; *MMSE*, Mini-Mental State Examination (score 0–9 = severe, 10–20 = moderate, 21–24 = mild, and 25–30 = normal); *ADL*, Activities of Daily Living Hierarchy Scale interRAI (0 = independent, 1 = supervision, 2 = limited, 3 = extensive, 4 = maximal, 5 = dependent, 6 = total dependency); *HADS*, Hospital Anxiety and Depression Scale (0–7 normal, 8–10 doubtful, 11 > definite).

^a^ 6 missing values, Sweden; ^b^11 missing values, Sweden; ^c^ 1 missing value, Sweden; ^d^ 3 missing values, Sweden

Participation was voluntary and signed informed consent was sought from the interested participants, both the persons with dementia and their caregiver, throughout the TECH@HOME and UP-TECH studies. If the individual could not provide consent due to reasons of being legally incompetent, substituted informed consent was sought from a family member or friend. The TECH@HOME study was approved by the Regional Ethical Review Board in Lund, Sweden (Dnr 2015/520), registered at ClinicalTrials.gov (NCT02733939). The UP-TECH project received approval from the Italian Regional Ethical Committee (Comitato Etico Regionale), and registered at ClinicalTrials.gov (NCT01700556) [38, 39].

In both studies, data was collected through structured questionnaires. The UP-TECH questionnaire included three sections, focusing on 1) general information about the person with AD, screening information and ethical evaluations; 2) caregiver’s demographics, socioeconomic status, lifestyle, clinical assessment, anthropometric measurements and caregiver relationship; and 3) dyad’s use of social and healthcare resources [38]. Similarly, TECH@HOME retrieved data on 1) persons with dementia’s sociodemographic information, cognitive function, ADL, quality of life and use of healthcare resources; and 2) caregiver’s sociodemographic information, lifestyle, quality of life, mental health and general wellbeing, caregiver burden, time spent caring and their use of healthcare services [39]. Both studies retrieved information on use of care resources made by the people with dementia using sections of the Resource Utilization in Dementia (RUD) instrument [46]. For this study, baseline data from both studies were used [38, 39]. Selected variables were categorised based on the Pearlin and colleagues’ model [11], which described the factors leading to outcomes of caregiving stress, including depression and anxiety. This model highlighted four main areas leading up to these outcomes, whereby 1) background and contextual circumstances of informal caregivers affected the 2a) primary and 2b) secondary stressors of dementia, which in turn were regulated by 3) mediators of stress [11].

In this study, self-reported symptoms of depression and anxiety were assessed using the Hospital Anxiety and Depression Scale (HADS) as the dependent variable [47, 48]. This instrument is composed by a set of 14 Likert scales of which seven are linked to depression and seven to anxiety. As each item is rated from 0 to 3, there was a total score ranging between 0 to 21; where, a cut-off score of four or more indicated symptoms of depression and a score of seven or more symptoms of anxiety. Hereby, depression and anxiety has a sensitivity of 0.864/0.938 respectively and a specificity of 0.788/0.847 respectively, indicating that the HADS is able to identify people with symptoms of depression or anxiety from those without [49]. Rather than having a cut-off score of 8 on both HADS scales for symptoms of depression and anxiety [50, 51], it is important that the HADS cut-points are reduced to consider all possible patients with mild psychiatric symptoms that are consistently distributed in any population [47, 49]. As independent variables, we used variables related to the:

- 1) *background circumstances* of the caregiver, such as if he/she lived with persons with dementia (yes/no); his/her physical health (in terms of number of diseases); gender; age; marital status; the level of education; and the type of relationship between the caregivers and persons with dementia (e.g. being a spouse, child or other type of relation).
- 2a) *primary stressors* of dementia, e.g. the stage of severity of the disease of the person with dementia, measured using the MMSE score categorized as severe (score of 0–9), moderate [10–21], mild [21–24] and normal [25–30] [52]; the occurrence of inappropriate behaviours (yes/no); the level of ADL dependence of the persons with dementia measured using the ADL Hierarchy interResident Assessment Instrument Contact Assessment (interRAI CA) [38, 39, 53]; and the total hours of caregiving provided by the caregivers during the day.
- 2b) *secondary stressors* of dementia related to the caregiver’s role: having an additional role as caregiver, an occupation, measured as actively working/ not actively working.
- 3) *mediators* that either worsen or ameliorate the development of symptoms of caregiver depression and anxiety, including the availability of additional caregiver support (yes/no) and the number of additional caregivers.

We used descriptive statistics, followed by simple and multiple forward step-wise linear regression analysis to test for associations between factors and the symptoms of depression and anxiety among the caregivers. The bivariate analysis allowed for a comparison between Sweden and Italy, as well as the exploration of differences in means and standard deviations (SD) of the independent variables, namely symptoms of depression and anxiety [54]. Statistical significance across each variable was determined using the Kruskal-Wallis (KW) test, where any *p*-values < 0.05 at 95% confidence interval (CI) were considered significant; and *p-*values < 0.2 were used for further analysis. Even though KW is a median test, for validity purposes, it was selected as a non-parametric back-up test, substituting ANOVA. This is because the dependent variables were not normally distributed but the difference of means was still intended to be explored [55, 56]. Variables associated with the two study outcomes (self-reported symptoms of depression and anxiety) at bivariate level with a *p*-value < 0.2 at 95% CI, were tested for independent association using linear regression models. Modelling was performed separately for Italy and Sweden. Any *p*-values < 0.05 at 95% CI were considered having significant associations with the dependent outcome variables. Multicollinearity and singularity were controlled for by testing each independent variable against another, via linear regression analysis [55]. For this analysis, specific categorical variables were coded into dummy variables, including age, education and type of relationship. This facilitated in representing, distinguishing and investigating between subgroup [54, 55].

Independent variables that had a *p*-value < 0.2 at 95% CI in the linear regression models were run against self-reported symptoms of depression and anxiety in additional multiple regression models. These independent variables were introduced one at a time in ascending order of significance, indicating any changes to adjusted R^2^, coefficient value, standard deviation (SD) and *p*-values. Additionally, a manual variable selection method via a forward step-wise elimination process was applied. With this, the entering of variables in the model was set at *p*-value < 0.20 at 95% CI and removal at *p*-value 0.05 at 95% CI. Overall, this analysis facilitated identifying the combination of factors associated with symptoms of depression and anxiety [55, 57]. All data treatment and further analysis were carried out using IBM SPSS Statistics 25 [58] and Microsoft Excel 2010.

## Results

The results of the bivariate analysis are represented in Tables 2 and 3, indicating the relationships between the independent variables selected according to Pearlin and colleagues’ model [11], and self-reported symptoms of depression and anxiety, across Sweden and Italy.

**Table 2:** Informal caregiver’s self-reported depression symptoms and the independent variables, between Italy and Sweden

Variables	Self-reported depression symptoms			
	Italy (***n*** = 317)		Sweden (***n*** = 89)	
	Mean (SD)	***p***	Mean (SD)	***p***
***Background and Context***				
**Gender** ^**a**^				
Male	6.28 (4.08)	< 0.001	3.43 (2.66)	0.419
Female	7.89 (4.32)		4.24 (3.53)	
**Age (years)** ^**a**^				
< 39	7.00 (4.55)	0.204	5.80 (5.50)	0.741
40–54	6.72 (4.19)		3.33 (2.63)	
55–69	7.49 (4.23)		4.18 (3.39)	
> 70	8.14 (4.45)		3.48 (2.65)	
**Marital status** ^**a**^				
Not Married	7.55 (4.28)	0.636	4.32 (3.64)	0.637
Married	7.35 (4.32)		3.69 (2.98)	
**Education** ^**a**^				
Elementary school	7.48 (4.14)	0.502	3.60 (3.06)	0.104
Gymnasium/ secondary	7.09 (4.63)		3.61 (3.42)	
University	7.96 (4.18)		5.23 (2.59)	
**Living with persons with dementia** ^**a b**^				
No	6.86 (4.20)	0.137	3.85 (3.45)	0.627
Yes	7.67 (4.35)		3.85 (2.78)	
**Physical health, disease count**				
0–2	7.39 (4.19)	0.626	3.82 (3.19)	0.544
3 or more	7.36 (5.07)		4.40 (2.51)	
**Type of relationship with persons with dementia** ^**a**^				
Wife/ husband (spouse)	8.16 (4.38)	0.095	3.97 (2.80)	0.616
Child	6.93 (4.25)		3.86 (3.42)	
Other	7.46 (4.22)		3.00 (3.52)	
***Primary Stressors of Dementia***				
**Severity of disease, MMSE** ^**d**^				
Normal (25+)	5 (1.41)	0.693	6 (n.a.)	0.150
Mild (21–25)	7.71 (4.54)		2.89 (2.20)	
Moderate (10–20)	7.38 (4.30)		4.23 (3.53)	
**Inappropriate behaviours** ^**e**^				
No	7.38 (4.43)	0.054	3.40 (2.86)	0.046
Yes	7.41 (3.96)		5.09 (3.54)	
**ADL dependency** ^**c**^				
0–2	7.23 (4.14)	0.328	3.84 (3.18)	–
3–6	8.07 (4.95)		–	
**Total hours of caregiving/ day** ^**f g**^				
≤ 5	7.49 (4.71)	0.573	3.54 (3.81)	0.098
≥ 6	7.01 (4.03)		3.83 (3.19)	
***Secondary Stressors of Dementia***				
**Occupation** ^**c**^				
Not actively working	7.37 (4.47)	0.730	4.10 (3.03)	0.188
Actively working	7.41 (4.12)		3.33 (3.01)	
***Mediators***				
**Additional caregiver support** ^**a**^				
No	8.27 (3.91)	0.033	4.09 (2.89)	0.247
Yes	7.18 (4.38)		3.61 (3.41)	
**Number of additional caregivers** ^**a**^				
0–1	7.23 (4.12)	0.387	3.78 (3.07)	0.835
2 or more	7.87 (4.81)		4.04 (3.43)	

Data source for Italy: UP-TECH questionnaire; Sweden: TECH@HOME questionnaire. Independent variables selected based on the Pearlin and colleagues’ model [11] (1990). *p-value* < 0.05 was regarded as significant; significant *p-values* are underlined.

*n*, number of participants; *SD*, standard deviation; *MMSE*, Mini-Mental State Examination (score 0–9 = severe, 10–20 = moderate, 21–24 = mild, and 25–30 = normal); *ADL*, Activities of Daily Living Hierarchy Scale interRAI (0 = independent, 1 = supervision, 2 = limited, 3 = extensive, 4 = maximal, 5 = dependent, 6 = total dependency); *HADS*, Hospital Anxiety and Depression Scale (0–7 normal, 8–10 doubtful, 11 > definite).

^a^ 2 missing values, Sweden; ^b^ 3 missing values, Italy; ^c^ 4 missing values, Sweden; ^d^ 6 missing values, Sweden; ^e^ 12 missing values, Sweden; ^f^ 17 missing values, Italy; ^g^ 5 missing values, Sweden

**Table 3:** Informal caregiver’s self-reported anxiety symptoms and the independent variables, between Italy and Sweden

Variables	Self-reported anxiety symptoms			
	Italy (***n*** = 317)		Sweden (***n*** = 89)	
	Mean (SD)	***p***	Mean (SD)	***p***
***Background and Context***				
**Gender** ^**a**^				
Male	5.80 (4.29)	0.002	2.31 (2.18)	0.299
Female	7.17 (4.10)		2.94 (2.63)	
**Age** ^**a**^				
< 39	7.71 (4.07)	0.777	3.80 (3.42)	0.486
40–54	6.51 (3.96)		2.19 (2.38)	
55–69	6.64 (4.11)		2.56 (2.53)	
> 70	7.05 (4.61)		2.85 (2.20)	
**Marital status** ^**a**^				
Not Married	6.27 (4.09)	0.293	3.18 (2.84)	0.341
Married	6.86 (4.23)		2.45 (2.27)	
**Education** ^**a**^				
Elementary school	6.88 (4.11)	0.528	2.49 (2.43)	0.066
Gymnasium/ secondary	6.50 (4.53)		2.39 (2.60)	
University	6.71 (3.38)		3.69 (1.80)	
**Living with persons with dementia** ^**a b**^				
No	6.53 (4.04)	0.616	2.44 (2.62)	0.175
Yes	6.88 (4.28)		2.87 (2.19)	
**Physical health, disease count** ^**a**^				
0–2	6.73 (4.13)	0.888	2.54 (2.44)	0.064
3 or more	6.81 (4.68)		4.20 (1.79)	
**Type of relationship with persons with dementia** ^**a**^				
Wife/ husband (spouse)	7.30 (4.46)	0.195	2.92 (2.25)	0.248
Child	6.55 (3.94)		2.52 (2.60)	
Other	6.26 (4.54)		1.67 (2.25)	
***Primary Stressors of Dementia***				
**Severity of disease, MMSE** ^**d**^				
Normal (25+)	2.00 (1.41)	0.175	5 (n.a.)	0.615
Mild (21–25)	7.62 (3.97)		2.58 (2.26)	
Moderate (10–20)	6.71 (4.20)		2.58 (2.53)	
**Inappropriate behaviours** ^**e**^				
No	6.75 (4.39)	0.781	2.51 (2.45)	0.430
Yes	6.70 (3.63)		3.00 (2.56)	
**ADL dependency** ^**c**^				
0–2	6.59 (4.15)	0.310	2.62 (2.44)	–
3–6	7.37 (4.40)		–	
**Total hours of caregiving/ day** ^**f g**^				
≤ 5	6.93 (4.22)	0.063	2.41 (2.34)	0.120
≥ 6	6.09 (4.03)		3.64 (2.85)	
***Secondary Stressors of Dementia***				
**Occupation** ^**c**^				
Not Actively Working	6.90 (4.11)	0.413	3.10 (2.36)	0.027
Actively Working	6.55 (4.31)		2.12 (2.38)	
***Mediators***				
**Additional caregiver support** ^**a**^				
No	6.98 (3.97)	0.416	3.19 (2.57)	0.044
Yes	6.68 (4.26)		2.09 (2.19)	
**Number of additional caregivers** ^**a**^				
0–1	6.62 (4.03)	0.615	2.75 (2.53)	0.689
2 or more	7.09 (4.68)		2.30 (2.16)	

Data source for Italy: UP-TECH questionnaire; Sweden: TECH@HOME questionnaire. Independent variables selected based on the Pearlin and colleagues’ model [11] (1990).

*p-value* < 0.05 was regarded as significant; significant *p-values* are underlined.

s*n*, number of participants; *SD*, standard deviation; *MMSE*, Mini-Mental State Examination (score 0–9 = severe, 10–20 = moderate, 21–24 = mild, and 25–30 = normal); *ADL*, Activities of Daily Living Hierarchy Scale interRAI (0 = independent, 1 = supervision, 2 = limited, 3 = extensive, 4 = maximal, 5 = dependent, 6 = total dependency); *HADS*, Hospital Anxiety and Depression Scale (0–7 normal, 8–10 doubtful, 11 > definite).

^a^ 2 missing values, Sweden; ^b^ 3 missing values, Italy; ^c^ 4 missing values, Sweden; ^d^ 6 missing values, Sweden; ^e^ 12 missing values, Sweden; ^f^ 17 missing values, Italy; ^g^ 5 missing values, Sweden

Generally, in this study sample, Italy showed a higher population percentage with symptoms of self-reported depression and anxiety (74 and 36% respectively) compared to Sweden (39 and 7% respectively). Therefore, even if the study sample average in the two samples is below the threshold of clinical significance, our analysis suggests the existence of subgroups of the caregivers’ population at a high risk for the two negative outcomes. With respect to the comparison of the average scores between the Swedish and Italian samples, we observed that all the differences were statistically significant (*p* < 0.05). For this reason in the following tables, we report only the results of the statistical testing between subgroups identified within (and not between) each national sample. Italian caregivers reported significantly higher symptoms of depression if they were female; this was also the case if they did not receive additional caregiver support. Swedish caregivers reported significantly more symptoms of depression if the persons with dementia showed inappropriate behaviour. Higher symptoms of anxiety were reported among Swedish caregivers, compared to those without, when they did not have an occupation alongside their caregiving role and had no additional caregiver for support. More self-reported symptoms of anxiety in Italy occurred among females. As mentioned, differences between Italy and Sweden when examining the relationship between the independent variables and the self-reported symptoms of depression and anxiety (Tables 2 and 3) were all statistically significant. For instance, the mean HADS depression score for females in Italy was 7.89; while in Sweden, the figures were 4.24 (Table 2). This pattern is detected for all variables for both HADS depression and HADS anxiety scores. In Italy, the female gender was significantly associated with greater self-reported symptoms of depression. When age was added to this model, the age group 40–54 years also showed a significant association (Table 4). In Sweden, only when the presence of additional caregivers was added to the model, age < 39 years, along with the total hours of caregiving per day showed an association with self-reported symptoms of depression, R2 = 0.193 (Table 4). When it comes to the factors associated with self-reported symptoms of anxiety, in Italy gender was strongly associated with the outcome along with the total hours of caregiving, once occupation and type of relationship were added to the model. Differently, in Sweden, the total hours of caregiving per day, additional caregivers and severity of disease had a strong association with the self-reported the symptoms of anxiety; R2 = 0.215 (Table 5).

**Table 4:** Multiple linear regression analysis for the independent variables and informal caregiver’s self-reported depression symptoms

Variables	Self-reported depression symptoms					
	Model 1		Model 2		Model 3	
	β Coefficient (SE)	***p***	β Coefficient (SE)	***p***	β Coefficient (SE)	***p***
***Italy (n = 316)***						
**Gender** (female vs. male)	1.607 (0.515)	< 0.001	1.810 (0.517)	< 0.001	−1.793 (0.516)	< 0.001
**Age, years** (ref. > 70)						
**< 39**			−0.836 (1.652)	0.613	−0.653 (1.649)	0.692
**40–54**			−1.703 (0.589)	0.004	−1.739 (0.587)	0.003
**55–69**			−0.863 (0.610)	0.158	−0.896 (0.608)	0.142
**Additional caregivers** (yes vs. no)					−1.091 (0.604)	0.072
**Constant**					6.183 (1.063)	< 0.001
**Adjusted R-squared changes**	0.027		0.043		0.050	
**R-squared**					0.065	
***Sweden (n = 86)***						
**Caregiving, hours/day** (0–24)	0.259 (0.078)	< 0.001	0.270 (0.078)	< 0.001	0.286 (0.077)	< 0.001
**Age, years** (ref. > 70)						
**< 39**			2.759 (1.444)	0.060	3.945 (1.569)	0.014
**40–54**			0.064 (0.862)	0.941	0.977 (0.989)	0.326
**55–69**			0.809 (0.762)	0.292	1.335 (0.807)	0.102
**Additional caregivers** (yes vs. no)					−1.362 (0.755)	0.075
**Constant**					2.517 (0.647)	< 0.001
**Adjusted R-squared changes**	0.104		0.120		0.143	
**R-squared**					0.193	

Data source for Italy: UP-TECH questionnaire; Sweden: TECH@HOME questionnaire.

*p-value* < 0.05 was regarded as significant; significant *p-values* are underlined.

*n,* number of participants; *β*, beta; *SE*, standard error; *CI*, confidence interval; *MMSE*, Mini-Mental State Examination (score 0–9 = severe, 10–20 = moderate, 21–24 = mild, and 25–30 = normal); *HADS*, Hospital Anxiety and Depression Scale (0–7 normal, 8–10 doubtful, 11 > definite).

**Table 5:** Multiple linear regression analysis for independent variables and informal caregiver’s self-reported anxiety symptoms in Italy and Sweden.

Variables	Self-reported anxiety symptoms							
	Model 1		Model 2		Model 3		Model 4	
	β Coefficient (SE)	p*	β Coefficient (SE)	p*	β Coefficient (SE)	p*	β Coefficient (SE)	p*
***Italy (n = 316)***								
**Gender** (female vs. male)	1.367 (0.504)	0.007	1.266 (0.511)	0.014	1.322 (0.510)	0.010	1.474 (0.518)	0.005
**Caregiving, hours/day** (≤5 vs. ≥6)			−0.795 (0.496)	0.110	−1.243 (0.549)	0.024	−1.205 (0.549)	0.029
**Occupation** (yes vs. no)					−0.988 (0.528)	0.062	−0.956 (0.527)	0.071
**Type of relationship** (ref. spouse)								
**Child**							−0.599 (0.534)	0.263
**Other**							−1.293 (0.758)	0.089
**Constant**							6.753 (1.283)	< 0.001
**Adjusted R-squared changes**	0.020		0.029		0.041		0.051	
**R-squared**							0.051	
***Sweden (n = 74)***								
**Caregiving, hours/day** (0–24)	0.142 (0.062)	0.025	0.148 (0.061)	0.017	0.164 (0.062)	0.010	0.148 (0.062)	0.019
**Additional caregivers** (yes vs. no)			−1.146 (0.497)	0.024	−1.133 (0.521)	0.033	−1.443 (0.539)	0.009
**Severity of disease, MMSE** (mild vs. moderate)					−1.181 (0.536)	0.031	−0.906 (0.547)	0.103
**Marital status** (yes vs. no)							1.136 (0.612)	0.068
**Constant**							3638 (0.837)	< 0.001
**Adjusted R-squared changes**	0.047		0.093		0.141		0.170	
**R-squared**							0.215	

Data source for Italy: UP-TECH questionnaire; Sweden: TECH@HOME questionnaire.

* *p-value* < 0.05 was regarded as significant; significant *p-values* are underlined

*n*, number of participants; *β*, beta; *SE*, standard error; *CI*, confidence interval; *MMSE*, Mini-Mental State Examination (score 0–9 = severe, 10–20 = moderate,

21–24 = mild, and 25–30 = normal); *HADS*, Hospital Anxiety and Depression Scale (0–7 normal, 8–10 doubtful, 11 > definite).

## Discussion

In general, our study showed that subgroups of the caregiver population might be at significant risk for self-reported anxiety and stress in Italy, where scores of HADS above 8 have been found among the caregivers who were spouses/partners, older than 70 years of age, caring for the most dependent ones, and who lack support from other informal caregivers.

With regards to the factors associated with HADS scores, our results confirm the findings of previous studies carried out in other EU countries [22, 37]. Our study showed that factors like gender, age, additional caregivers, total hours of caregiving, type of relationship and severity of dementia were associated with self-reported symptoms of depression and anxiety among dementia caregivers in Italy and Sweden.

In both countries, the number of total hours of caregiving in this study was associated with anxiety levels; in Italy when adjusted for occupation and in Sweden when adjusted for additional caregivers and disease severity. Italian caregivers spend many hours in caregiving due to their cultural and social norms [31]. If they have an occupation additional to their caregiving role they tend to experience less family support, ultimately increasing their risk of developing symptoms of anxiety [59]. In this study, Swedish caregivers experienced increased self-reported symptoms of anxiety and depression when they spent more than 6 h of caregiving per day. This appears to be due to the increased disease severity they face in later stages of dementia, and the lack of additional support from formal or informal caregivers. When examining the total hours of caregiving, it is crucial to understand the role of the caregiver within the different stages of a dementia disease. This may be because in the early stages of dementia, when the persons with dementia only require simple surveillance, the understanding of total hours of caregiving is different compared to the later stages of dementia, that require more demanding, protective and active engagement of the caregivers.

Additional caregivers are helpful and play an important role in both countries to reduce the symptoms of depression among caregivers; which could be observed by the changes in the R^2^ values when ‘additional caregivers’ was added into the linear regression model. However, the type of caregiving support varies across the two countries. While caregivers in Italy require more formal support to complement the role of the family, Swedish caregivers seek more tailored emotional and practical support that can provide family-support management, particularly in later stages of the dementia disease [18, 22].

In fact, our results highlight significant differences between and within countries regarding self-reported depression and anxiety of informal caregivers for persons with dementia. In Italy, females and persons aged between 40 and 54 years were more likely to show self-reported symptoms of depression. Italian caregivers who had an additional occupation and/or were female were also more likely to show self-reported symptoms of anxiety. Meanwhile, in Sweden, age (< 39 years) and more than 6 h of caregiving per day were associated with self-reported symptoms of depression, and fewer additional caregivers and disease severity with self-reported symptoms of anxiety. Additionally, our findings generally show that Italian caregivers of persons with dementia report more symptoms of depression and anxiety compared to caregivers in Sweden.

There are potential explanations for this cross-country difference, which also highlights the complexity of the issue. Firstly, Italian caregivers often lack support in terms of formal services, home-care and professional training from the government [18, 31, 35]. This shortcoming of support might increase the risks of caregivers reporting symptoms of depression and anxiety, especially among those who cannot afford the limited services available [18]. Conversely, Sweden is known to have greater availability of services as the municipalities are obliged by law to support caregivers [34]. Secondly, Italian caregivers are more likely to live with their care recipients, consequently spending more hours in caregiving compared to Swedish caregivers. As they find less time for themselves and are more exposed to physical, emotional and financial constraints, they are more likely to experience psychological distress [19]. Thirdly, Italians tend to avoid talking about psychological problems and turn to self-reliance as a coping strategy, experience some form of stigmatisation and develop a negative attitude towards the help from a health professional who is not a psychologist [60]. In Sweden, the public and local government instead tend to show more positive attitudes towards people with caregiver burden and provide more accepted social and health services respectively [61].

Our findings demonstrated that among Italian caregivers’ gender and age were associated with symptoms of depression, especially when adjusted for additional caregivers. Older female Italian caregivers, aged 40–54 years, were more likely to experience symptoms of depression. They may be at higher risk as they additionally bear the responsibility for caring after their children as well as managing their two-part role as a caregiver and worker [8]. However, it cannot be assumed that women are more emotionally vulnerable to the changing health state of the persons with dementia compared to men since the psychological strain of caregiving is a very complex matter. In fact, women may place very high responsibility and expectations on their caregiving role, that when they are not met, they show more self-reported symptoms of depression [8]. Whilst living at home with the care recipient can provide them with additional family support and help with coping strategies, it also means that they spend more hours on average in caregiving compared to men or Swedish caregivers, which may increase caregiver burden and consequently symptoms of depression and anxiety [19]. Thus, these findings not only confirm that older people and women tend to be more prone to psychological distress [62], but that there are still gender disparities amongst caregivers in Italy. Due to their cultural and social norms, particularly Italian female caregivers are expected to care for the persons with dementia from home [31]. Conversely, in this study sample, Sweden does not show an association between gender and symptoms of psychological distress. On the one hand this cannot confirm findings of previous studies conducted in different European countries [16], on the other hand, some studies carried out in Sweden also found no significant correlation between the caregiver’s gender and burden [63, 64]. This potentially reflects the present success of the Swedish welfare system’s objective, namely improving and providing equal rights for employment, education, social and health services, particularly amongst women [31].

In Sweden, the factors more strongly associated with self-reported symptoms of depression were age (< 39 years) and total hours of caregiving, especially when adjusted for the variables controlling for the availability of an additional caregivers. Not only are the majority of dementia caregivers in Sweden working adult children [63], but they also show more symptoms of depression, compared to the usual predicted people group, namely spouses, who generally provide more hours of caregiving [65]. The young and working caregivers may develop feelings of guilt as they are unable to provide adequate care as they struggle in reconciling their care-work balance [59, 64]. Additionally, they may experience difficulty in accepting the caregiving role and understanding dementia due to their lack of experience and knowledge of the progression of the disease, especially in later stages of dementia when the level of dependency of the persons with dementia and total hours of caregiving increase. Consequently, young and working informal caregivers tend to be overwhelmed and more likely to experience caregiver burden, such as psychological distress [27, 64].

The severity of disease of the persons with dementia, measured as cognitive impairment, had a higher coefficient of association with symptoms of anxiety in Sweden than in Italy, therefore suggesting a stronger relation with this clinical dimension. There may be a lack of knowledge and education among the caregivers on the consequences of the dementia progression and related caregiving difficulties which could increase the symptoms of anxiety and fear of the future [66]. In contrast, Italian caregivers may be more prepared through support and information provided by their cohabiting family members. In fact, it is proven that family support plays a vital role in improving the caregiver’s quality of life [63]. Since Swedish caregivers often lack family support [22, 59], the severity of dementia of a person with dementia continues to be associated with the caregiver’s increased experience of isolation, distress and particularly anxiety, confirming findings of previous studies [22].

In Italy, the main factor associated with the severity of anxiety symptoms was gender. The association was particularly strong when adjusted for type of relationships with the person with dementia. Once again, this emphasises the existence of gender disparities in Italy among dementia caregivers [31].

Even though a strength of cross-sectional studies like this study is the ability to generalise the findings into other contexts [67], it is also important to be aware of the possible differences in level of perceived psychological distress of dementia caregivers across as well as within countries [22, 59]. Both, the TECH@HOME study and the UP-TECH-study, recruited participants from only one region of Sweden and Italy respectively. In addition, due to the nature of a cross-sectional study it may be difficult to infer causality. However, this was accounted for in this study by the investigation of correlations between variables only, rather than cause-effect relationships, due to the baseline data that were used. Due to the use of the database originating from TECH@HOME and UP-TECH [38, 39], the data could not enable investigation of all potential variables of interest. This might limit the findings as a consequence of a possible ecological bias. We could not explore quality of life and health of the caregivers (physical and psychological); quality of relationship with persons with dementia; years of caregiving experience; and variables representing the intrapsychic secondary stressors of the Pearlin and colleagues’ model [11], i.e. personality traits of the caregivers like level of self-esteem, neuroticism, confidence in the caregiver role and coping strategies [8]. Last but not least, despite the accurate sampling process and the careful recruitment process, the risk of the typical sampling bias should still be mentioned, as for instance, healthier and more educated older caregivers are more likely to participate in research studies (especially those involving technologies, such as TECH@HOME and UP-TECH [68]. Nonetheless we have minimized the likelihood of such risk, adjusting our analysis using available socio-economic variables.

## Conclusions

Informal dementia caregiver self-reported symptoms of depression and anxiety are a complex matter; not only are numerous factors involved, but they also differ across various healthcare and welfare states. It is important to investigate these variables in future research to identify risk groups and fully comprehend the factors associated with symptoms of depression and anxiety experienced by caregivers of persons with dementia. The understanding of these factors cross-culturally and their association with symptoms of depression and anxiety among informal caregivers is vital for future planning and policy-making purposes when designing cost-effective interventions and EU-wide strategies for all caregivers of persons with dementia. Only then, the quality of intervention programs and ultimately the health of each informal caregiver of persons with dementia can be improved in a time where dementia and caregiver’s self-reported symptoms of depression and anxiety are a growing public health concern.

## Data Availability

The baseline dataset used and analysed during this study is available from the corresponding author on reasonable request.

## Abbreviations

AD: Alzheimer’s disease
ADL: Activities of Daily Living
CI: Confidence interval
DSM-5: Diagnostic and Statistical Manual of Mental Disorders
EU: European Union
GDP: gross domestic product
GDS: Global Deterioration Scale
HADS: Hospital Anxiety and Depression Scale
HIC: High Income Country
ICT: Information and Communication Technology
interRAI CA: interResident Assessment Instrument Contact Assessment
KW: Kruskal-Wallis
MMSE: Mini-Mental State Examination
MMSE-SR: Mini-Mental State Examination Svensk Revidering
NIA-AA: National Institute on Ageing-Alzheimer’s Association
OECD: Organisation for Economic Co-operation and Development
RUD: Resource Utilization in Dementia
SD: standard deviation
